# 2.5-Minute Fast Brain MRI with Multiple Contrasts in Acute Ischemic Stroke

**DOI:** 10.1007/s00234-024-03331-0

**Published:** 2024-03-11

**Authors:** Annika Kits, Jonathan Al-Saadi, Francesca De Luca, Fredrik Janzon, Michael V. Mazya, Johan Lundberg, Tim Sprenger, Stefan Skare, Anna Falk Delgado

**Affiliations:** 1 Department of Clinical Neuroscience, Karolinska Institutet, Stockholm, Sweden; 2 Department of Neuroradiology, Karolinska University Hospital, Solna, 17176 Stockholm, Sweden; 3 Department of Radiology, Karolinska University Hospital, Stockholm, Sweden; 4 Department of Radiology, Danderyd Hospital, Stockholm, Sweden; 5 Department of Neurology, Karolinska University Hospital, Stockholm, Sweden; 6 MR Applied Science Laboratory Europe, GE Healthcare, Stockholm, Sweden

**Keywords:** Fast imaging, DWI/T2-FLAIR mismatch, Multi-contrast MRI, NeuroMix, Ischemic stroke, Diagnostic performance

## Abstract

**Purpose:**

To assess the performance of a 2.5-minute multi-contrast brain MRI sequence (NeuroMix) in diagnosing acute cerebral infarctions.

**Methods:**

Adult patients with a clinical suspicion of acute ischemic stroke were retrospectively included. Brain MRI at 3 T included NeuroMix and routine clinical MRI (cMRI) sequences, with DWI/ADC, T2-FLAIR, T2-weighted, T2*, SWI-EPI, and T1-weighted contrasts. Three radiologists (R1–3) independently assessed NeuroMix and cMRI for the presence of acute infarcts (DWI ↑, ADC = or ↓) and infarct-associated abnormalities on other image contrasts. Sensitivity, specificity, and the area under the receiver operating characteristic curve (AUC) were calculated and compared using DeLong’s test. Inter- and intra-rater agreements were studied with kappa statistics. Relative DWI (rDWI) and T2-FLAIR (rT2-FLAIR) signal intensity for infarctions were semi-automatically rendered, and the correlation between methods was evaluated.

**Results:**

According to the reference standard, acute infarction was present in 34 out of 44 (77%) patients (63 ± 17 years, 31 men). Other infarct-associated signal abnormalities were reported in similar frequencies on NeuroMix and cMRI (*p* > .08). Sensitivity for infarction detection was 94%, 100%, and 94% evaluated by R1, R2, R3, for NeuroMix and 94%, 100%, and 100% for cMRI. Specificity was 100%, 90%, and 100% for NeuroMix and 100%, 100%, and 100% for cMRI. AUC for NeuroMix was .97, .95, and .97 and .97, 1, and 1 for cMRI (DeLong *p* = 1, .32, .15), respectively. Inter- and intra-rater agreement was *κ* = .88–1. The correlation between NeuroMix and cMRI was *R* = .73 for rDWI and *R* = .83 for rT2-FLAIR.

**Conclusion:**

Fast multi-contrast MRI NeuroMix has high diagnostic performance for detecting acute cerebral infarctions.

**Supplementary Information:**

The online version contains supplementary material available at 10.1007/s00234-024-03331-0.

## Introduction

Fast echo-planar imaging (EPI)-based single-scan brain MRI technique EPIMix [1] renders multiple MRI contrasts (DWI/ADC, T2-FLAIR, T2-weighted, T2*, and T1-weighted) with 4–5-mm slice thickness. Despite a short acquisition time of just over 1 min, high diagnostic performance for acute cerebral infarction has been reported [2, 3]. Recently, this technique has been further developed to improve resolution and signal-to-noise ratio while reducing geometric distortions [4] by replacing EPI with motion-insensitive [5] single-shot fast spin echo (SSFSE)-based T2-weighted and T2-FLAIR and adding three-dimensional SWI-EPI and T1-weighted EPI within a total acquisition time below 3 min. NeuroMix provides seven different contrasts (DWI/ADC, SSFSE T2-weighted, SSFSE T2-FLAIR, T2*, T1-weighted, 3D SWI-EPI, 3D T1-EPI) in one continuous scan.

NeuroMix can be useful in clinical situations where fast imaging is necessary and patients may have difficulty lying still [6], like suspected ischemic stroke [7]. In acute ischemic stroke, the reperfusion treatment time window extends to the first 24 h after symptom onset if salvageable brain tissue exists [7–9], depending on reperfusion strategy (thrombolysis or thrombectomy) and findings on imaging [10], and may extend even beyond 24 h [11]. Imaging has to be fast [7] because earlier reperfusion treatment improves patient outcomes [12]. MRI is the most sensitive imaging method for detecting the ischemic core [13], with abnormal high signal appearing on DWI within the first minutes [14] and on T2-FLAIR within the first hours, with a gradual increase of lesion visibility after symptom onset [15, 16]. Emphasizing the benefits of the NeuroMix multi-contrast approach, the simultaneous generation of other contrasts besides DWI and T2-FLAIR enables the exclusion of hemorrhage (SWI, T2*, T2-weighted) [17], which is a prerequisite for thrombolysis [7]. Previous reports on fast single-scan multi-contrast MRI have focused on patients outside the reperfusion treatment window [2, 3] but need to be confirmed in an acute stroke cohort since early ischemic lesions can be more challenging to detect on both DWI and T2-FLAIR [13, 15, 18–21]. Furthermore, it is unclear how improvements in the image quality of NeuroMix [4] will affect the findings.

Thus, the primary aim was to evaluate the diagnostic performance of the fast multi-contrast brain MRI sequence NeuroMix against the reference standard for detecting acute infarction on DWI in patients with suspected stroke and MRI performed early, within the first 12 h from initial CT evaluation. Secondary aims assessed intra- and inter-rater agreements compared to clinical MRI (cMRI) and infarction-associated signal changes on other image contrasts (T2-FLAIR, SWI, T2-weighted, T2*, and T1-weighted).

## Materials and methods

The study was approved by the Swedish ethical review authority, waiving the need for informed consent.

### Participants

The picture archiving and communication system (PACS) was searched for retrospective inclusion of consecutive adult patients with a radiological stroke work-up at the Karolinska University Hospital between May 2021 and August 2022. Eligible cases were patients with acute multimodal computed tomography (CT) referrals from inpatient and emergency departments with a clinical suspicion of acute ischemic stroke and a subsequently performed acute MRI of the brain. Exclusion criteria were the timing of MRI more than 12 h after initial brain CT, missing NeuroMix sequence, or missing DWI/ADC on standard MRI. NeuroMix had previously been included at our center as a motion-robust addition to the cMRI protocol analyzed when routine images were uninterpretable due to motion. Hence, every eligible examination included both cMRI and NeuroMix. cMRI was acquired before NeuroMix.

Clinical data were collected from the radiology referrals and medical charts.

### Image acquisition

Scans were acquired on a Signa Premier 3 T (GE Healthcare, WI, USA) with a 48-channel brain or 19-channel head-neck coil. The acquisition time for NeuroMix was 2.5 min and 5.5–13.5 min for cMRI, depending on the included sequences in the selected clinical protocol. Table 1 presents the acquisition time and order of each sequence contrast. All of the NeuroMix sequence contrasts were acquired as one continuous scan, which included five motion-robust 2D contrasts (single-shot EPI sequences of DWI, T1-FLAIR EPI and T2* gradient echo EPI, and single-shot fast spin echo sequences of SSFSE T2-weighted and SSFSE T2-FLAIR), as well as two optional 3D contrasts (3D SWI-EPI and 3D T1-weighted-EPI), which lack motion-robust properties. Out of these contrasts, DWI, 3D T1-weighted-EPI, and 3D SWI-EPI were acquired in a sequential manner as separate entities, while T1-FLAIR EPI, T2* gradient echo EPI, SSFSE T2-weighted, and SSFSE T2-FLAIR were run in two passes for odd and even slices. More technical details of NeuroMix have been previously described [4].

**Table 1:** Sequence parameters of NeuroMix and cMRI and number of available cases for each contrast. The sequence contrasts are shown in the fixed order of acquisition for NeuroMix, while the order was variable for cMRI

MRI contrast	Acquisition time (s)	Scan plane	Matrix size	Slice thickness (mm)	FOV (mm)	Pixel size (mm2)	Number of cases with available contrast (*n*/%)
NeuroMix							
3D T1-weighted EPI *	23	Sagittal	192 × 192	1.2	240 × 240	1.2 × 1.2	44/100%
DWI spin echo EPI	33.5	Axial	180 × 180	4	240 × 240	1.3 × 1.3	44/100%
T1-FLAIR EPI	16.8	Axial	180 × 180	4	240 × 240	1.3 × 1.3	44/100%
T2* gradient echo EPI	2	Axial	180 × 180	4	240 × 240	1.3 × 1.3	44/100%
T2-weighted SSFSE	15.4	Axial	240 × 180	4	240 × 180	1.0 × 1.0	44/100%
T2-FLAIR SSFSE	23.3	Axial	220 × 136	4	240 × 180	1.1 × 1.3	44/100%
3D SWI EPI *	28	Axial	312 × 312	2	240 × 240	0.8 × 0.8	44/100%
cMRI							
3D SWI EPI	45	Axial	320 × 320	2	240 × 240	0.8 × 0.8	44/100%
3D T1-weighted fast spin echo	287	Sagittal	320 × 320	1	256 × 256	0.8 × 0.8	1/2%
DWI spin echo EPI	129	Axial	192 × 192	4	240 × 240	1.3 × 1.3	44/100%
T2* gradient echo EPI	NA	NA	NA	NA	NA	NA	0
T2-weighted turbo spin echo propeller	189	Axial	380 × 380	3	240 × 240	0.6 × 0.6	34/77%
3D T2-FLAIR fast spin echo	153	Sagittal	272 × 272	1.6	240 × 240	0.8 × 0.8	44/100%

*cMRI* routine clinical MRI, *DWI* diffusion-weighted imaging, *EPI* echo-planar imaging, *FLAIR* fluid-attenuated inversion recovery, *FOV* field of view, *mm* millimeter, *NA* not available, *NeuroMix* multi-contrast brain MRI, *s* seconds, *SSFSE* single-shot fast spin echo, *SWI* susceptibility weighted imaging, *3D* three dimensional

*This image contrast is an optional part of NeuroMix sequence [4]

### Image evaluation

Anonymized NeuroMix and cMRI images were rated in PACS by three readers. Reader 1 (FDL) was a radiologist in training with 4 years of experience reading brain MRI; readers 2 (FJ) and 3 (AK) were neuroradiologists with 13 and 19 years of experience, respectively. Readers were aware of the clinical suspicion of ischemic stroke but blinded to other clinical information. The NeuroMix data were read first, followed by a memory washout interval of at least 2 weeks. Readers were pre-trained on an online training tool for the DWI and T2-FLAIR mismatch rating [6].

All readers assessed the presence of acute infarction on DWI/ADC (high signal on DWI and low or iso signal on ADC). The reference standard for the main study outcome acute infarction diagnosis was established as follows:

1. A congruent diagnosis among all three readers (readers 1–3) on both NeuroMix and cMRI.
2. In cases of disagreement, a diagnosis was reached by additional consensus reading of the three readers by re-evaluating all available images from both methods.

In detail, image assessment on NeuroMix and cMRI DWI/ADC, T2-FLAIR, T2-weighted, SWI, T2*, and T1-weighted was performed according to Supplemental Table 1.

Artifacts were assessed by reader 3, and cases with artifacts were not excluded from the analysis.

### Quantitative analysis

NeuroMix and cMRI images were processed using Matlab 2021b, FMRIB Software Library, and Freesurfer 7.3.2. Brain extraction was performed using the Freesurfer SynthStrip tool. Semi-automatic segmentation was used to identify acute infarctions. An initial mask was created using an ADC threshold of 620 µm^2^/s. Voxels without neighboring voxels and voxels without a signal above one standard deviation of the mean DWI signal were excluded. Infarction masks were inspected by reader 3, blinded to clinical information, and manually corrected to remove false positive mask voxels obviously outside the DWI lesion area (i.e., artifacts near the skull base and voxels on slices outside the infarcted area were removed). Voxels near the infarcted area were not corrected. No voxels were manually added to the mask. The DWI images and infarction masks were coregistered to the T2-FLAIR using FMRIB’s Linear Image Registration Tool and to the MNI space atlas using FMRIB’s Nonlinear Image Registration Tool to identify infarction locations automatically. The signal intensity in the infarction masks was divided by non-infarction voxels ± 2 slices around the infarction to calculate the relative DWI (rDWI) and relative T2-FLAIR (rFLAIR) signal. Tissue-type segmentation was performed using FMRIB’s Automated Segmentation Tool to remove CSF from the non-infarction voxels.

### Statistical analysis

The diagnostic performance for acute infarction was evaluated by calculating the area under the receiver operating characteristic curve (AUC), sensitivity, specificity, and accuracy for NeuroMix compared to the reference standard for each reader. These calculations were similarly performed for cMRI compared to the reference standard. The AUC for NeuroMix and cMRI were compared using DeLong’s test [22]. A comparison of the number of cases with abnormal signal on T2-FLAIR, T2-weighted, SWI, and T2-FLAIR/DWI mismatch between NeuroMix and cMRI was performed with McNemar’s test. Inter-rater agreement was evaluated with Fleiss’ and intra-rater agreement with Cohen’s Kappa. Infarction sizes, rDWI, and rFLAIR values were compared using linear correlation analysis and the Spearman correlation coefficient. *p*-values < 0.05 were considered statistically significant. Statistical analysis was performed in NCSS Statistical Software (2021), Matlab (The Mathworks Inc., R2021b), and MedCalc^Ⓡ^ (19.3.1 MedCalc).

## Results

**Fig. 1:**
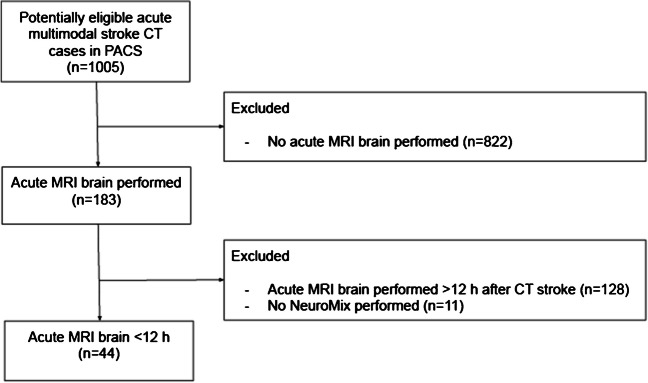
Flow diagram

Acute infarctions were present in 34 out of 44 (77%) included patients (mean age, 63 ± 17 [SD] years, 31 men), Fig. 1. An overview of clinical symptoms, cardiovascular risk factors, recanalization therapy, and imaging delay is presented in Table 2.

**Table 2:** Demographic and clinical characteristics

Age years, mean ± SD	63 ± 17
Female	30%
Acute infarction present	34/44 (77%)
Cerebrovascular disease risk factors present	36/44 (82%)
Presenting NIHSS, mean ± SD	9 ± 6
Reperfusion therapy performed before MRI	16/44 (36%)
Reperfusion therapy performed after MRI	9/44 (9%)
Infarction volume (ml) range, median (IQR)	0.0–62.3, 1.6 (0.4–9.1)
Patients with known symptom onset	25/44 (56%)
Patients with unknown symptom onset	11/44 (26%)
Patients with wake-up stroke	8/44 (18%)
Patients with a delay from symptom onset to MRI < 4.5 h	6/44 (14%)
Delay from symptom onset to MRI (h) range (median; IQR)	2–44 (7; 5–11)

*IQR* interquartile range, *ml* milliliter, *NIHSS* NIH Stroke Scale, *SD* standard deviation

**Fig. 2:**
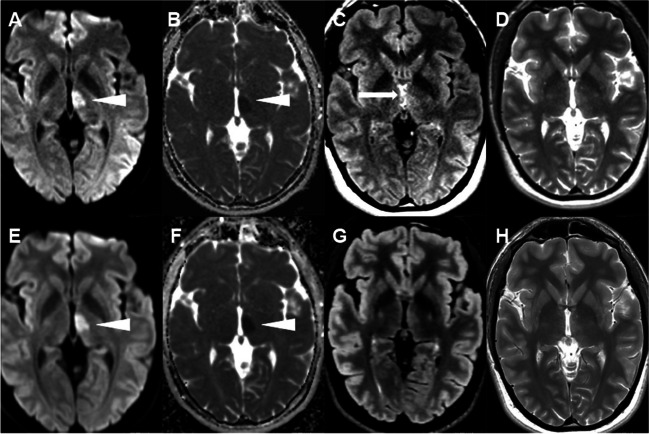
Images in a 27-year-old man who presented with right-sided weakness and dysphasia (NIHSS 6) at wake-up. CT without arterial occlusion. MRI was performed to detect DWI/T2-FLAIR mismatch. Axial DWI, ADC, T2-FLAIR, and T2-weighted images, **A**–**D** NeuroMix upper row and **E**–**H** cMRI lower row. **A, E** A high-signal DWI lesion with a corresponding low signal on **B, F** ADC is visible in the left thalamus with similar conspicuity on NeuroMix compared to cMRI (arrowhead). **C, D, G, H** On T2-FLAIR and T2-weighted images, a subtle high signal on NeuroMix and cMRI is present in the DWI lesion area with low conspicuity on both methods. T2-FLAIR abnormality is slightly more visible on cMRI due to a higher signal-to-noise ratio on cMRI and **C** adjacent high-signal cerebrospinal fluid pulsation artifacts in the third ventricle that are present only on NeuroMix T2-FLAIR (arrow). The clinical radiological interpretation was acute infarction with DWI/T2-FLAIR mismatch, and thrombolytic treatment with intravenous alteplase was given after MRI with a reduction of symptoms (NIHSS from 6 to 2)

The reference standard for acute infarction was established by congruent assessment of all three readers on NeuroMix and cMRI in 41/44 (93%) and by consensus diagnosis of the three readers in 3 of 44 (7%) patients. For NeuroMix, all its image contrasts were available, while DWI, T2-FLAIR, and SWI-EPI data were available for all patients on cMRI (Table 1). Time from symptom onset to MRI was known in 25 of 44 (56%) patients with a median delay of 7.1 h (range 2.0–44.0, IQR 4.6–11.1). MRI exams within 4.5 h from symptom onset were performed in 6 out of 44 (14%) patients, while 8 out of 44 (18%) patients had wake-up stroke. The primary clinical indication for MRI was confirmation of infarction diagnosis in 28/44 (64%), assessment of DWI/T2-FLAIR mismatch in 8/44 (18%) (Fig. 2), and description of extension of the infarction in 8/44 (18%) of the cases.

### Main outcome diagnostic performance

**Fig. 3:**
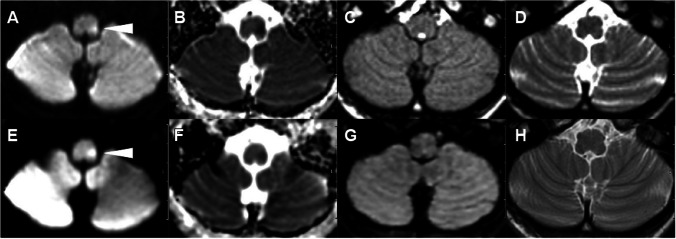
Images in a 55-year-old woman who presented with left-sided weakness and posterior circulation symptoms (NIHSS 10). CT without infarction but thrombosis of the left vertebral artery was shown on CT angiography. Axial DWI, ADC, T2-FLAIR, and T2-weighted images, **A**–**D** NeuroMix upper row and **E**–**H** cMRI lower row. **A, E** MRI performed 2.9 h after symptom onset shows a subtle DWI lesion to the left in the medulla oblongata (arrowhead). The DWI lesion has lower conspicuity on NeuroMix compared to cMRI. All readers rated the lesion as negative on T2-FLAIR corresponding to DWI/T2-FLAIR mismatch. The clinical radiological interpretation was acute infarction with DWI/T2-FLAIR mismatch, and treatment with alteplase was given after MRI with a reduction of symptoms (NIHSS from 10 to 2)

**Fig. 4:**
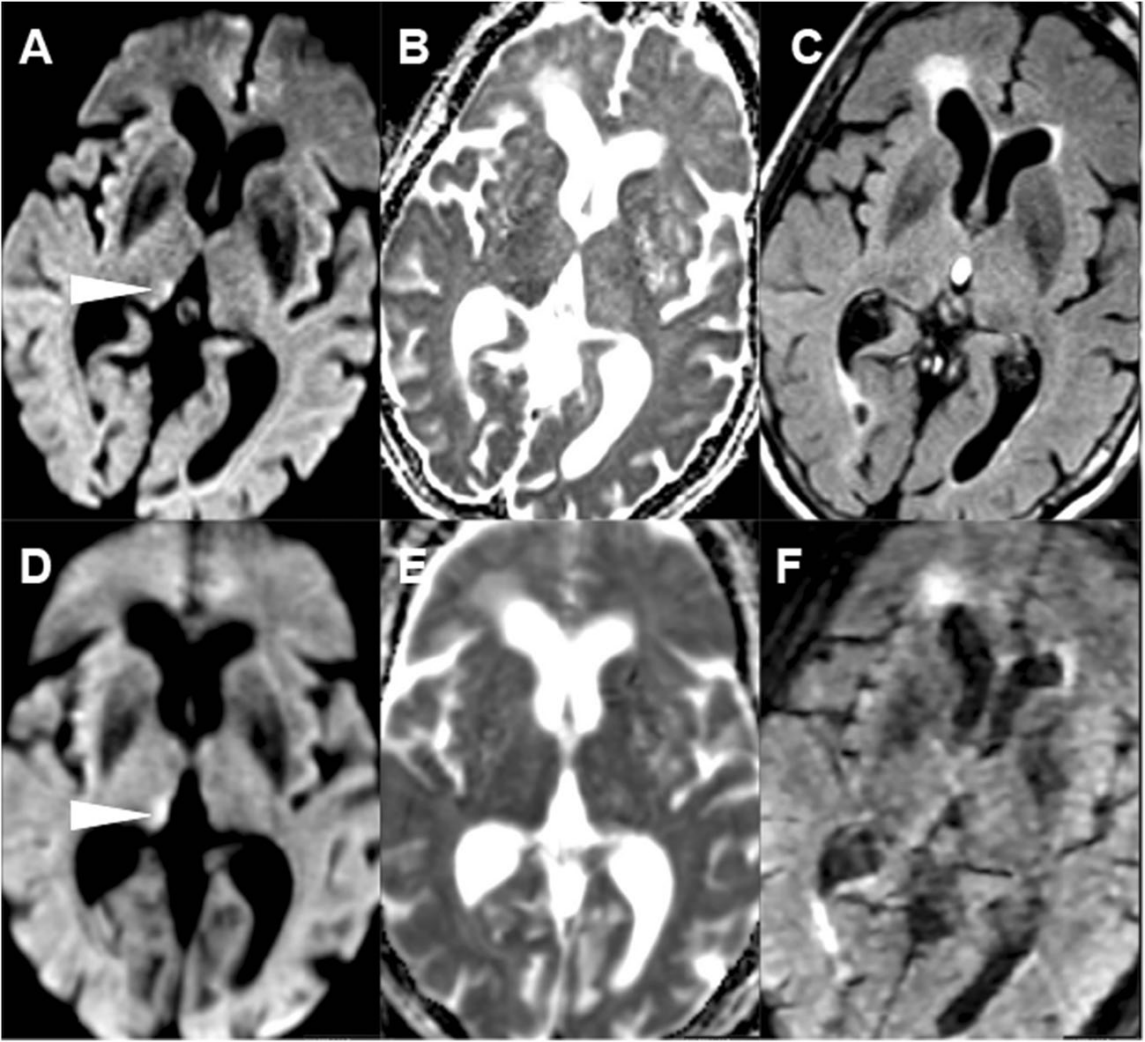
Images in an 80-year-old woman with left-sided weakness (NIHSS 24) and unknown onset of symptoms. CT without infarction or arterial occlusion on CT angiography. MRI was performed 1 h after CT to confirm infarction diagnosis. Axial DWI, ADC, and T2-FLAIR images, **A**–**C** NeuroMix upper row and **D**–**F** cMRI lower row. The patient could not lie still, resulting in motion artifacts, mainly on **F** cMRI T2-FLAIR and to a lower extent on **D** cMRI DWI. **A**, **D** A faint high-signal lesion is visible on DWI in the medial thalamus on the right side on both NeuroMix and cMRI (arrowhead). The lesion was classified as acute infarction by consensus reading. The lesion is not visible on NeuroMix T2-FLAIR, and cMRI T2-FLAIR was impossible to analyze due to motion artifacts. Notably, the clinical radiological reading detected no lesion with the interpretation that no infarction was present, reperfusion treatment was not administered, and the evolution of NIHSS was not documented

Sensitivity for detecting an abnormal DWI lesion on NeuroMix by the three readers was 94%, 100%, and 94%, and specificity was 100%, 90%, and 100% compared to the reference standard. Sensitivity for an abnormal DWI lesion on cMRI was 94%, 100%, and 94%, while specificity was 100% for all three readers. No relevant difference of AUC between NeuroMix (0.95, 0.98, and 0.95) and cMRI AUC (0.97, 1.00, and 1.00) was found (*p* = 1.00, 0.32, 0.15 DeLong). Detailed results of diagnostic performance (sensitivity, specificity, accuracy, AUC values, DeLong’s test of AUC values, and intra-rater agreement) per reader for NeuroMix and cMRI are presented in Table 3. The inter-rater agreement was high for detecting acute infarction for both NeuroMix (*κ* = 0.88 [95% CI 0.83–0.92]) and cMRI (*κ* = 0.92 [95% CI 0.87–0.96]). There were a total of six false-negative ratings in two cases, four false negatives for NeuroMix (R1 and R3), and two for cMRI (R1). Both false negative cases had low conspicuity of DWI lesions: one case with a short (2.9 h) delay from stroke symptom onset to MRI with an infarction in the medulla oblongata (Fig. 3) and a case with wake-up stroke where MRI was performed 8.9 h after last known well with infarction in the right thalamus (Fig. 4). In the one false-positive rating, a focal cortical artifact of subtle high signal on DWI NeuroMix was incorrectly interpreted as infarction by one reader (R2), Supplemental Fig. 1.

**Table 3:** Diagnostic performance for acute infarction detection compared to reference standard

	NeuroMix R1	cMRI R1	NeuroMix R2	cMRI R2	NeuroMix R3	cMRI R3
TP	32	32	34	34	32	34
FP	0	0	1	0	0	0
FN	2	2	0	0	2	0
TN	10	10	9	10	10	10
Sensitivity (%) (95% CI)	94 (80–99)	94 (80–99)	100 (90–100)	100 (90–100)	94 (80–99)	100 (90–100)
Specificity (%) (95% CI)	100 (69–100)	100 (69–100)	90 (56–100)	100 (69–100)	100 (69–100)	100 (69–100)
Accuracy	.95	.95	.98	1.00	.95	1.00
AUC (95% CI)	.97 (.89–. 99)	.97 (.92–.99)	.95 (.68–.99)	1.00	0.97 (.89–. 99)	1.00
Pairwise comparison of AUC NeuroMix versus cMRI (*p*) DeLong	1		.32		.15	
Intra-rater agreement NeuroMix versus cMRI κ (95% CI)	1		.93 (.80–1.00)		.88 (.72–1.00)	

*AUC* area under the curve, *CI* confidence interval, *cMRI* routine clinical MRI, *FN* false negative, *FP* false positive, *κ* kappa (Cohen, agreement: 0–.20 slight, .20–.40 fair, .41–.60 moderate, .61–.80 substantial, .81–1.00 almost perfect), *NeuroMix* multi-contrast brain MRI, *R1* reader 1, *R2* reader 2, *R3* reader 3, *TN* true negative, TP true positive

### Secondary outcomes

The presence of an abnormal signal in the infarcted area on T2-FLAIR, T2-weighted, and SWI images, as well as the presence of DWI/FLAIR mismatch, CMB, and asymmetric prominent vessel sign on SWI with comparisons between NeuroMix and cMRI, is presented in Table 4 with the number of cases with abnormal findings for each reader in Supplemental Fig. 2. No relevant differences were found between NeuroMix and cMRI (*p* > 0.08). T1-weighted and T2* sequences were unavailable for cMRI (Table 1) and were not further analyzed.

**Table 4:** Secondary outcomes: abnormal findings on NeuroMix and cMRI on each image contrast with corresponding agreement statistics

Evaluation	NeuroMix number of cases with abnormal findings for R1–3 (mean; SD)	cMRI number of cases with abnormal findings for R1–3 (mean; SD)	*p* (McNemar) for R1, R2, and R3	Intra-rater agreement NeuroMix versus cMRI for R1–3 (Cohen’s) κ	Inter-rater agreement NeuroMix (Fleiss) κ (95% CI)	Inter-rater agreement cMRI (Fleiss) κ (95% CI)
1 a	26; 2.6	29; 2.1	.17, .26, .27	.79, .80, .77	.69 (95% CI .65–.72)	.78 (95%CI .74–.82)
1 b	10; 5.3	7; 2.9	.69, .39, .17	.70, .76, .69	.54 (95% CI .51–.57)	.62 (95% CI .59–.66)
2	19; 4.9	20; 4.2	.41, .32, .50	.83, .94, .72	.60 (95% CI .56–.64)	.61 (95% CI .57–.65)
3	9; 1.7	10; 1.0	.08, .56, .56	.80, .81, .78	.67 (95% CI .63–.72)	.57 (95% CI .52–.61)
4	23; 5.1	20; 4.2	.41, .26, .41	.73, .68, .71	.51 (95% CI .47–.55)	.54 (95% CI .49–.58)
5	10; 4.6	9; 5.5	.20, .31, .58	.38, .60, .35	.27 (95% CI .22–.31)	.18 (95% CI .14–.21)
6*	19; 9.5	NA	NA	NA	.46 (95% CI .43–.50)	NA
7*	8; 1.2	NA	NA	NA	.69 (95% CI .66–.72)	NA

There was no significant difference (*p* > 0.05) between NeuroMix and cMRI for any of the detected abnormal findings

*1 a* presence of abnormal high T2-FLAIR signal, *1 b* presence of DWI/T2-FLAIR mismatch, *2* abnormal high T2 signal in the DWI lesion area, *3* abnormal low signal on SWI in the DWI lesion area representing intra-infarct hemorrhage, *4* abnormal small foci of low signal on SWI in any part of the brain representing cerebral microbleed, *5* SWI asymmetric prominent vessel sign, *6* abnormal low T1 signal in the DWI lesion area, *7* abnormal low signal on T2* in the DWI lesion area representing intra-infarct hemorrhage, *CI* confidence Interval, *cMRI* routine clinical MR, *κ* kappa (0–.20 slight, .20–.40 fair, .41–.60 moderate, .61–.80 substantial, .81–1.00 almost perfect), *NA* not available, *NeuroMix* multi-contrast brain MRI, *R1* reader 1, *R2* reader 2, *R3* reader 3

*Contrast available only on NeuroMix

### Quantitative analysis

**Fig. 5:**
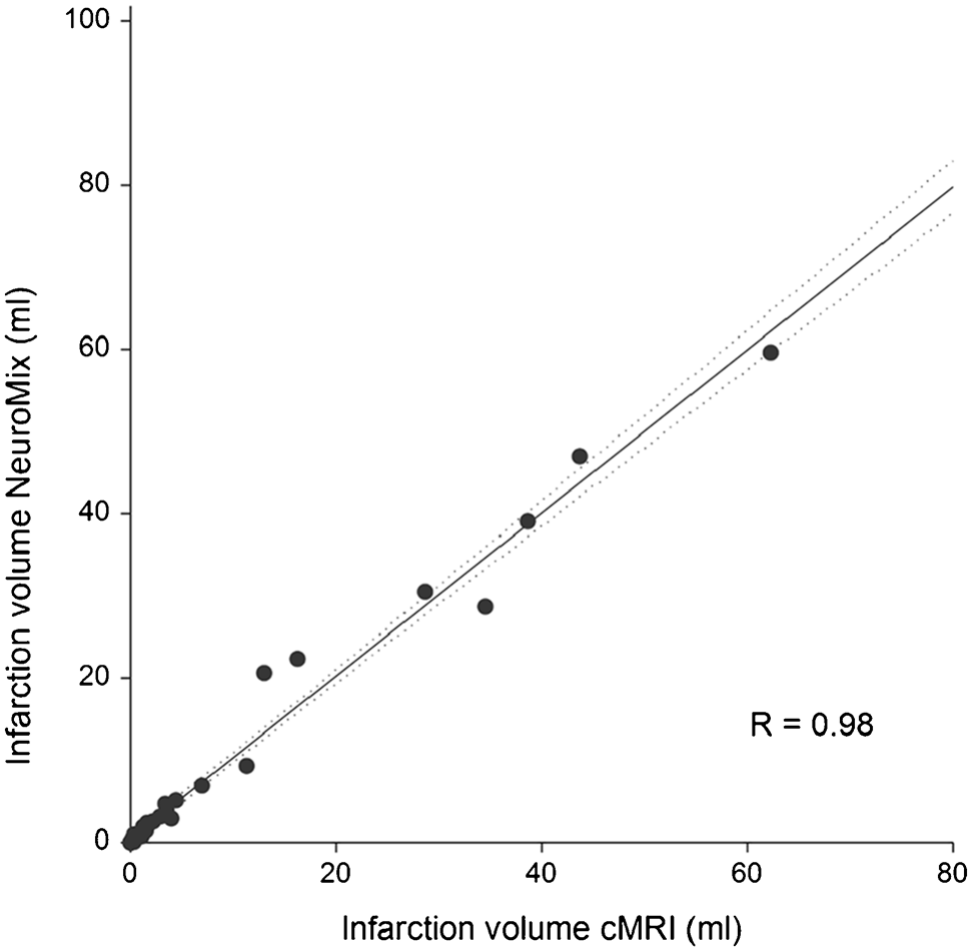
Infarction sizes (ml) on NeuroMix and cMRI from semi-automatic segmentation. Each dot represents a single case. The solid line represents the fit with dotted lines representing confidence bounds (95%). *R*, Spearman correlation coefficient

**Fig. 6:**
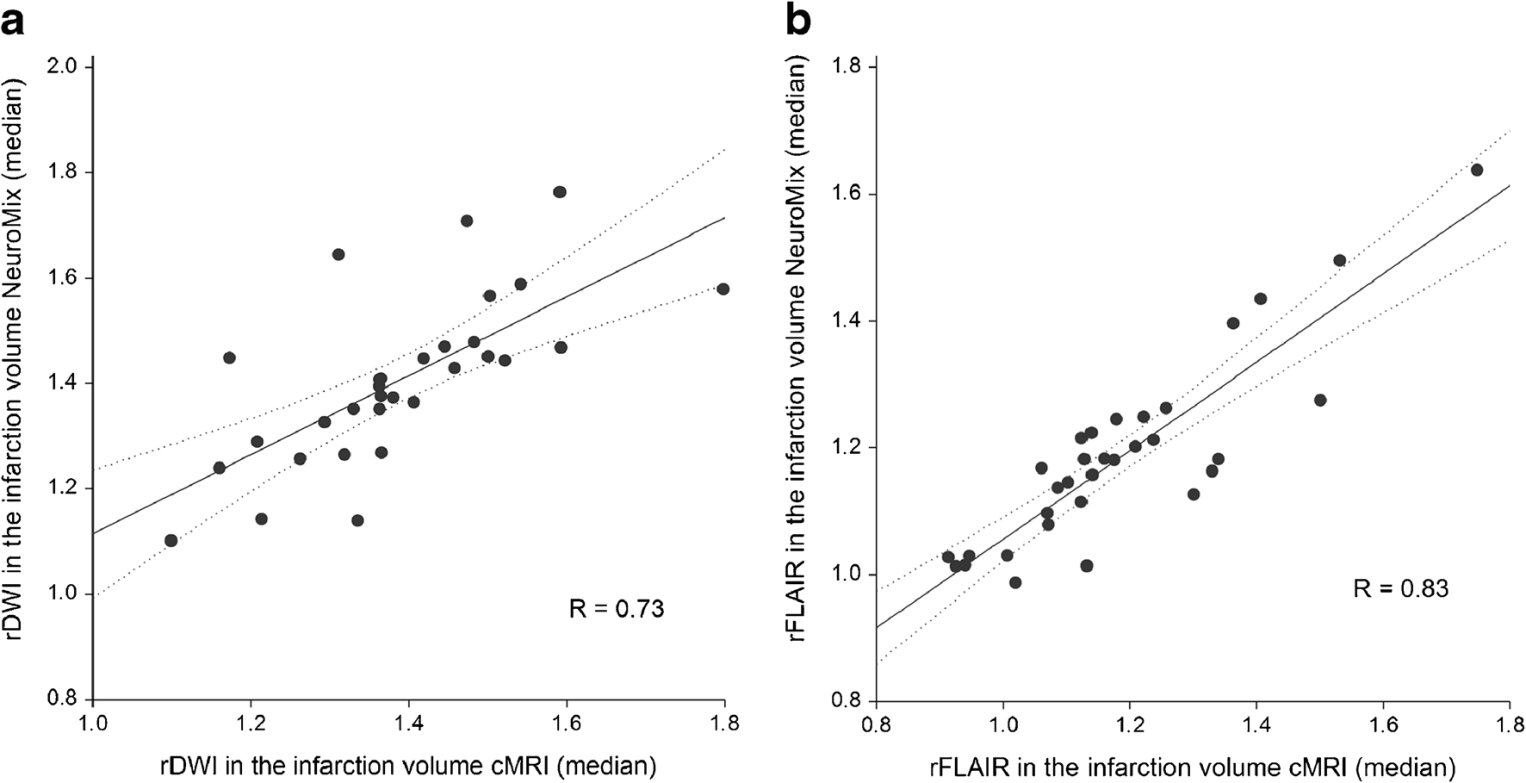
Linear regression analysis of **A** relative DWI signal intensity (rDWI) and **B** relative T2-FLAIR signal intensity (rFLAIR) on NeuroMix and cMRI in the infarcted area compared to surrounding normal brain tissue for each case in semi-automatic segmentation (*n* = 32). Each dot represents a single subject. The solid line represents the fit with dotted lines representing confidence bounds (95%). *R*, Spearman correlation coefficient

Semi-automatically rendered infarction size was 0.1–59.6 ml (median 2.3, IQR 0.7–8.7) for NeuroMix and 0.0–62.4 ml (median 1.6, IQR 0.4–9.1) for cMRI, *R* = 0.98 (95% CI 0.96–0.99), *p* = 0.04 (Fig. 5). Both false positive and false negative infarction mask voxels were present in both methods, with completely missed infarcted area in two cases for NeuroMix (Figs. 3 and 4) and in one case for cMRI (Fig. 4). The anatomical locations of infarctions are presented in Supplemental Table 2. rDWI in infarcts was 1.1–1.8 (median 1.4, IQR 1.2–1.5) for NeuroMix compared with 1.1–1.8 (median 1.4, IQR 1.3–1.5) for cMRI (*p* = 0.5), with a correlation coefficient of 0.73 (95% CI 0.51–0.86), Fig. 6A. rT2-FLAIR was 1.0–1.6 (median 1.2, IQR 1.1–1.2) for NeuroMix and 0.9–1.7 (median 1.1, IQR 1.1–1.3) for cMRI (*p* = 0.4), with a correlation coefficient of 0.83 (95% CI 0.68–0.91), Fig. 6B.

### Artifacts

Image artifacts were present in four cases, potentially affecting the infarction diagnosis.

In two cases, artifacts related to hardware (one coil-related and one RF-related issue) caused low signal-to-noise on both NeuroMix and cMRI. In one case, a problem with the calibration scan resulted in bilateral frontal signal void on NeuroMix, while cMRI was unaffected. Lastly, motion artifacts were present in one case affecting cMRI on T2-FLAIR and DWI, but not NeuroMix, Fig. 4.

## Discussion

This study evaluated the diagnostic performance of a 2.5-min brain MRI technique called NeuroMix for the detection of acute infarction on DWI in patients presenting with acute stroke symptoms. Out of 44 adult patients (63 ± 17 years, 31 men), acute infarctions were present in 34 cases (77%). The diagnostic performance for NeuroMix was high, with a sensitivity of 94–100% and a specificity of 90–100%. High sensitivity and specificity in stroke detection have been reported previously for its predecessor technique EPIMix, with sensitivity > 90% and specificity > 98% [2, 3]. NeuroMix could achieve high diagnostic performance in stroke despite including patients with a relatively short time from symptom onset to MRI (median 7.1 h) in the current study. These results are in line with previously reported fast imaging protocols that have been tested in patients with ischemic stroke [2, 3, 23–25]. DWI has a reported sensitivity of 90–100% and specificity of 75–100% on standard MRI [21, 26], as was also observed in our study with a sensitivity of 94–100% and a specificity of 100% for routine clinical MRI (cMRI). Notably, both cMRI and NeuroMix DWI had imaging parameters similar to those used in many stroke centers [13, 20, 27]. The lack of evidence for a difference in infarction diagnosis on NeuroMix compared to cMRI (AUC 0.95–0.97 versus AUC 0.97–1.00 *p* > 0.15) was further supported by the quantitative analysis showing a strong correlation between NeuroMix and cMRI for infarction size (*R* = 0.98), and signal changes on rDWI (*R* = 0.73). The two false-negative cases can be related to more difficult detection of DWI abnormality in infarctions with short symptom duration, small size, and posterior fossa location [15, 20, 28].

Besides DWI, other standard contrasts useful in stroke evaluation are generated during one image acquisition from NeuroMix without prescans or scan operator interactions between series. The duration of NeuroMix (2.5 min) was shorter compared to cMRI (5.5 min for DWI/ADC, T2-FLAIR, and SWI or 13.5 min with the addition of T2-weighted and T1-weighted) and compared to around 13 min for stroke protocols in other centers [29]. It is important to compare NeuroMix to model-based brain imaging techniques such as synthetic MRI [30] and MRI fingerprinting [31]. Common for the latter is that they are more time consuming and first acquire parametric proton density and T1 and T2 maps, from which weighted T1 and T2 MRI contrasts can be produced. Recently, simultaneous visualization of cerebral vasculature with post-processed synthetic MRI phase-sensitive inversion recovery images has been reported [32], while NeuroMix lacks angiographic sequence. However, it is not possible to synthesize DWI or SWI/T2* from the model-based parametric maps, currently limiting their application in stroke. A pulse sequence able to simultaneously acquire DWI and T2* contrasts has been developed [33], but this technique does not include T2-FLAIR. Deep-learning algorithms that generate synthetic T2-FLAIR from DWI with a time reduction of the MRI protocol have shown similar diagnostic performance to T2-FLAIR [34].

In NeuroMix, SSFSE T2-FLAIR renders better image quality and lacks EPI-related geometric distortions compared to EPI T2-FLAIR while maintaining fast and motion-robust properties [4]. Despite a lower resolution and signal-to-noise ratio on NeuroMix SSFSE T2-FLAIR, there was a strong correlation between NeuroMix and cMRI rT2-FLAIR (*R* = 0.83) in the infarcted area. Further, there was no evidence of a difference between NeuroMix and cMRI for the detection of T2-FLAIR hyperintense lesions (*p* > 0.17) and DWI/T2-FLAIR mismatch (*p* > 0.17). The intra-rater agreement was substantial (*κ* > 0.69) for detection of both T2-FLAIR high signal and DWI/T2-FLAIR mismatch in the infarcted area in line with previous fast protocols [23, 34]. The inter-rater agreement was substantial for detecting abnormal T2-FLAIR high-signal lesions. However, the inter-rater agreement was only moderate for DWI/T2-FLAIR mismatch on NeuroMix, while it was substantial for cMRI. A poor-to-moderate agreement for subjective DWI/T2-FLAIR mismatch rating has been previously reported [15, 16, 27, 35, 36]. NeuroMix’s similar detection of pathology compared to cMRI T2-FLAIR suggests the potential of using it as a diagnostic tool to detect even subtle T2-FLAIR lesions like infarctions in the reperfusion treatment window. In one case, cMRI had disturbing motion artifacts on DWI and T2-FLAIR, while NeuroMix was not affected. This highlights the potential of NeuroMix to reduce motion-related issues that can occur in patients with suspected stroke [6]. The five 2D sequences of NeuroMix possess inherent motion robust properties due to the ability of fast single-shot EPI and FSE to freeze motion, thus enabling artifact-free images even in cases when motion sensitive 3D SWI-EPI and 3D T1-weighted-EPI may be disturbed [4]. Due to the NeuroMix sequence design where different contrasts were acquired as separate entities, patient motion during some part of the imaging only affects the specific contrasts when motion occurs.

One limitation of the study is the single-center retrospective design. As a sequence-related limitation, NeuroMix is an in-house developed pulse sequence compatible with one manufacturer. While the multi-contrast sequence allows for a complete assessment of lacunar infarcts, and when selecting patients for thrombolysis, it is important to note that NeuroMix currently does not include perfusion and angiography for stroke imaging. Adding these contrasts may increase the acquisition time, if CT angiography and CT perfusion have not been performed previously. In the study, NeuroMix included 2D T2* gradient echo EPI and SWI, along with 2D T1-FLAIR EPI and 3D T1-weighted EPI, which may result in contrast redundancy. Notably, the 3D contrasts are optional, while the 2D contrasts are a fixed part of the NeuroMix. Therefore, the 3D contrasts can be excluded and imaging time allocated for other sequences, such as angiographic or perfusion sequences, to optimize imaging and patient care depending on the clinical context.

## Conclusion

The fast multi-contrast brain MRI pulse sequence had a high diagnostic performance for detecting acute infarction on DWI, with similar detection of associated signal changes on other image contrasts, as routine clinical MRI.

### Supplementary Information

Below is the link to the electronic supplementary material.

Supplementary file1 (DOCX 1400 KB)

Supplementary file2 (DOCX 23.2 KB)

## Data Availability

The data presented in this study are available from the corresponding author, upon reasonable request.

## Abbreviations

ADC: Apparent diffusion coefficient
AUC: Area under the receiver operating characteristic curve
cMRI: Routine clinical MRI
DWI: Diffusion-weighted imaging
FLAIR: Fluid-attenuated inversion recovery
NeuroMix: Multi-contrast brain MRI
NIHSS: National Institutes of Health Stroke Scale
R1: Reader 1
R2: Reader 2
R3: Reader 3
