# Supplementary material for: 2.5-Minute Fast Brain MRI with Multiple Contrasts in Acute Ischemic Stroke
Source: Neuroradiology. 2024 Mar 11;66(5):737–47. doi: 10.1007/s00234-024-03331-0 (PMC11031482; doi:10.1007/s00234-024-03331-0)
Supplement: Supplementary file 1 — Supplementary file1 (DOCX 1400 KB) [file 234_2024_3331_MOESM1_ESM.docx]

**Supplemental Figure 1**


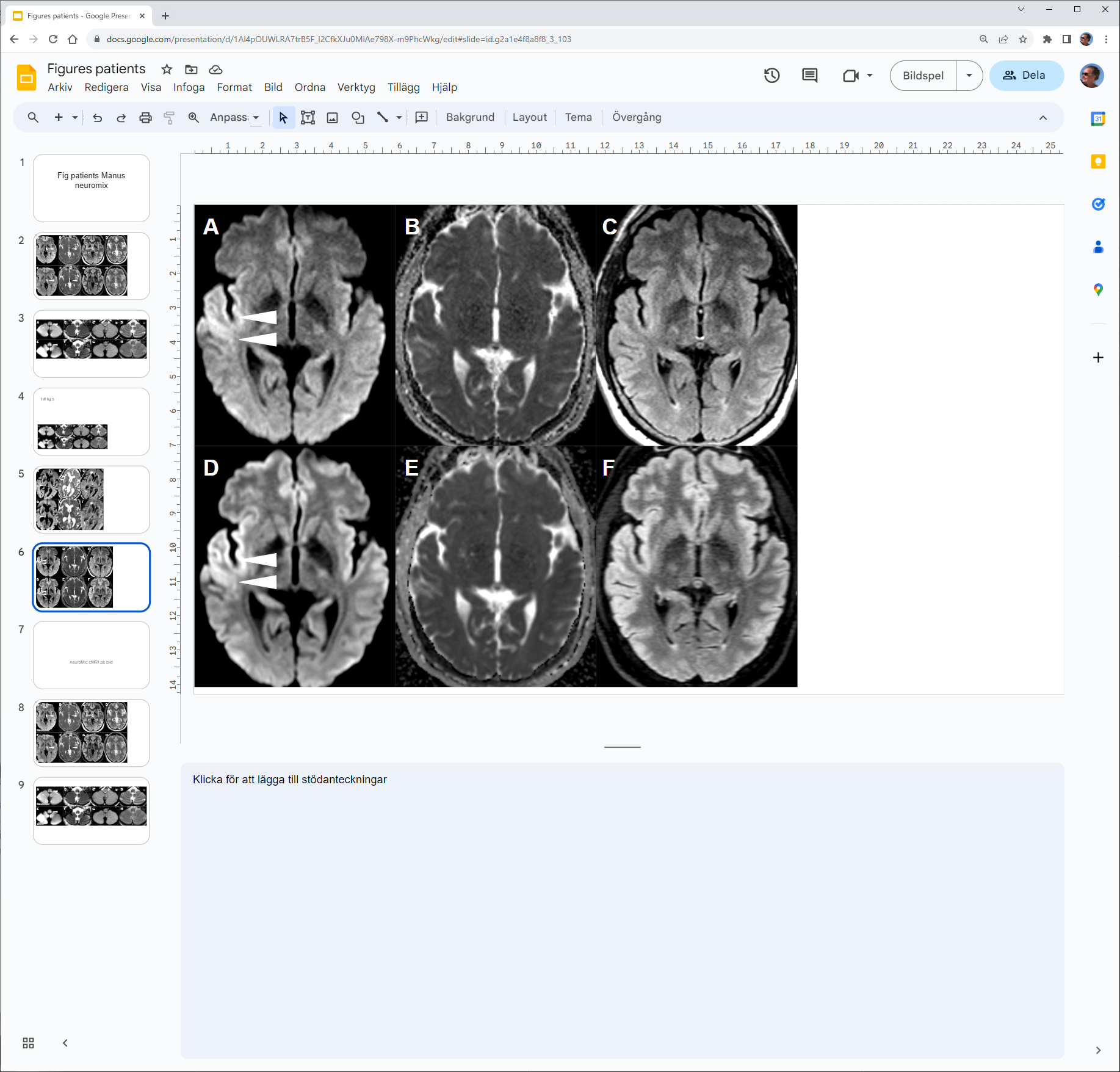
Images in a 39-year-old man with a false positive rating who presented with a right-sided weakness (NIHSS 2). No infarction or arterial occlusion on CT. MRI was performed 5 hours after symptom onset to confirm infarction diagnosis. Axial DWI, ADC, and T2-FLAIR images, (**A-C**) NeuroMix upper row, (**D-F**) cMRI lower row.

One reader incorrectly rated a cortical-subcortical artifact of subtle high signal in the right temporal lobe on (**A**) NeuroMix DWI (arrowheads) as infarction (false positive rating). On consensus reading no infarction was detected. Although a similar high signal in the right temporal lobe is present on (**D**) cMRI DWI (arrowheads), it was correctly interpreted on cMRI as a normal finding. A possible explanation for the interpretation of infarction on NeuroMix might be due to a slight hypointensity in this area on NeuroMix ADC compared to cMRI ADC due to more noise on NeuroMix ADC. The clinical radiological interpretation was that there is no infarction present and no treatment was administered.

**Supplemental Figure 2**


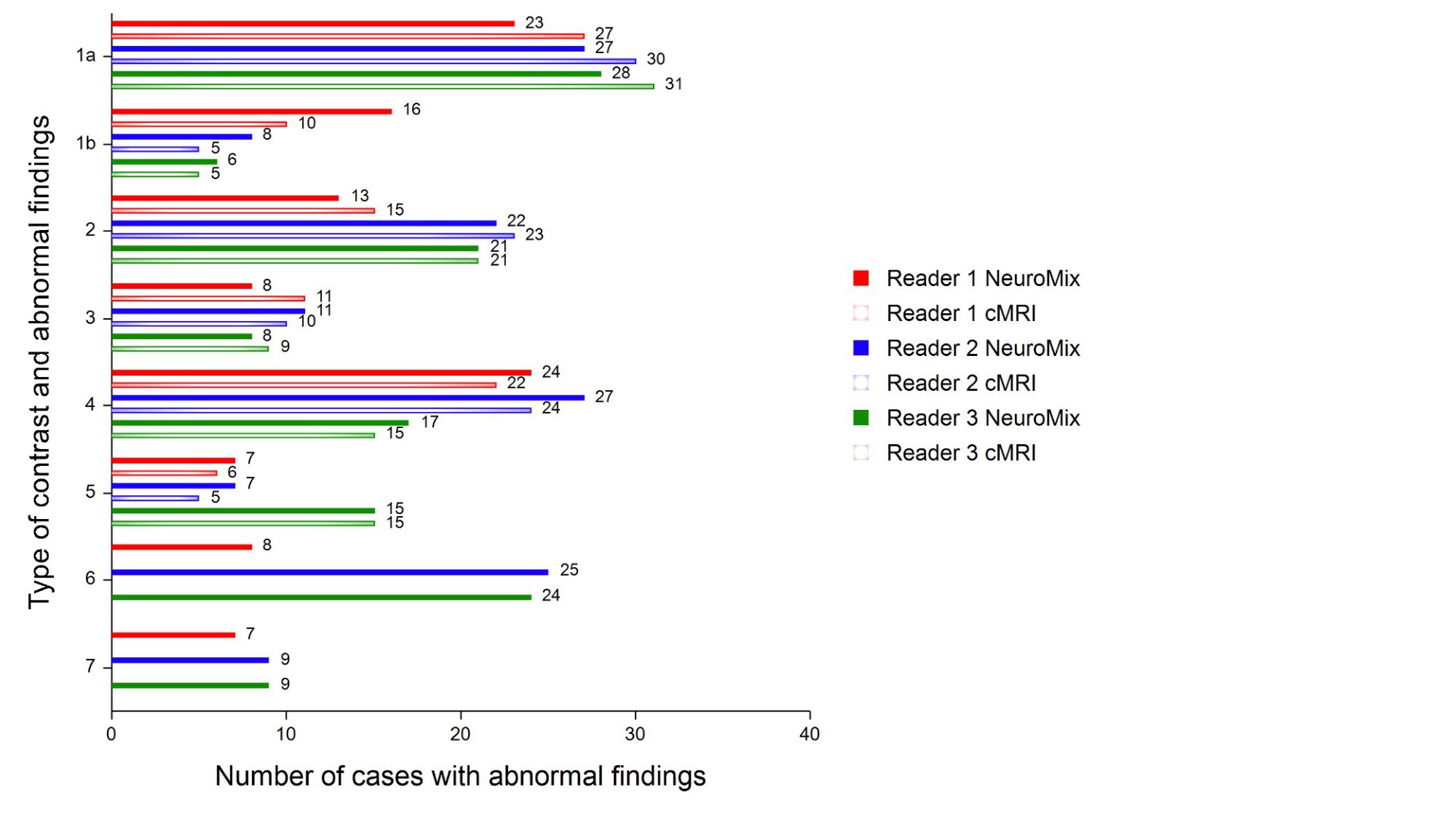
Number of abnormal findings on NeuroMix and cMRI on each image contrast for individual readers. Type of image contrast and abnormal findings: 1 a, Presence of abnormal high T2-FLAIR signal; 1 b, Presence of DWI/T2-FLAIR mismatch; 2, Abnormal high T2 signal in the DWI lesion area; 3, Abnormal low signal on SWI in the DWI lesion area representing intra-infarct hemorrhage; 4, Abnormal small foci of low signal on SWI in any part of the brain representing cerebral microbleed; 5, SWI asymmetric prominent vessel sign; 6, Abnormal low T1 signal in the DWI lesion area (T1-weighted was available only for NeuroMix); 7, Abnormal low signal on T2* in the DWI lesion area representing intra-infarct hemorrhage (T2* was available only for NeuroMix)

There was no significant difference between NeuroMix and cMRI for any of the detected abnormal findings (p >.08).
