# Supplementary material for: 2.5-Minute Fast Brain MRI with Multiple Contrasts in Acute Ischemic Stroke
Source: Neuroradiology. 2024 Mar 11;66(5):737–47. doi: 10.1007/s00234-024-03331-0 (PMC11031482; doi:10.1007/s00234-024-03331-0)
Supplement: Supplementary file 2 — Supplementary file2 (DOCX 23.2 KB) [file 234_2024_3331_MOESM2_ESM.docx]

**2.5-Minutes Fast Brain MRI with Multiple Contrasts in Acute Ischemic Stroke**

**Supplemental Table 1.** **Secondary outcomes, image assessment on NeuroMix and cMRI**

| Evaluation number | Evaluated contrast | Abnormal finding | Rating |
| --- | --- | --- | --- |
| 1* | T2-FLAIR* | Abnormal high T2-FLAIR signal in the DWI lesion area | 1.1 Clear T2-FLAIR high signal  1.2 Subtle T2-FLAIR high signal  1.3 No T2-FLAIR high signal  1.4 Assessment not appropriate due to disturbing artifacts or leukoaraiosis in the lesion area |
| 1 a |  | Presence of abnormal high T2-FLAIR signal | 1. Presence of abnormal T2-FLAIR signal (rating 1.1 and 1.2)  2. No T2-FLAIR high signal (rating 1.3)  3. Assessment not appropriate (rating 1.4) |
| 1 b |  | Presence of DWI/T2-FLAIR mismatch** | 1. Presence of DWI/T2-FLAIR mismatch (rating 1.2 and 1.3)  2. No DWI/T2-FLAIR mismatch (rating 1.1)  3. Assessment not appropriate (rating 1.4.) |
| 2 | T2-weighted | Abnormal high T2 signal in the DWI lesion area | 1. Yes  2. No |
| 3 | SWI | Abnormal low signal on SWI in the DWI lesion area representing intra-infarct hemorrhage | 1. Yes  2. No |
| 4 | SWI | Abnormal small foci of low signal on SWI in any part of the brain representing cerebral microbleeds (CMB) | 1. Yes  2. No |
| 5 | SWI | Asymmetric prominent vessel sign*** | 1. Yes  2. No |
| 6 | T1-weighted | Abnormal low T1 signal in the DWI lesion area | 1. Yes  2. No |
| 7 | T2* | Abnormal low signal on T2* in the DWI lesion area representing intra-infarct hemorrhage | 1. Yes  2. No |

*For further statistical analysis the four T2-FLAIR categories (1.1‒4.1) were regrouped to evaluate:

1 a. Presence of abnormal T2-FLAIR signal

1 b. Presence of DWI/T2-FLAIR mismatch

******Acute ischemic lesion visible on DWI (“positive DWI”) but no marked parenchymal hyperintensity visible on T2-FLAIR (“negative T2-FLAIR”) represents “DWI/T2-FLAIR mismatch” (Thomalla G, Simonsen CZ, Boutitie F, Andersen G, Berthezene Y, Cheng B, et al. MRI-Guided Thrombolysis for Stroke with Unknown Time of Onset. N Engl J Med [Internet]. 2018 Aug 16;379(7):611–22. Available from: <http://dx.doi.org/10.1056/NEJMoa1804355>**)**

*******Abnormally increased number of hypointense vessels or vessel caliber compared to contralateral hemisphere on SWI inside or outside of the abnormal DWI lesion area represents “asymmetric prominent vessel sign” (Jiang HF, Zhang YQ, Pang JX, Shao PN, Qiu HC, Liu AF, et al. Factors associated with prominent vessel sign on susceptibility-weighted imaging in acute ischemic stroke. Sci Rep [Internet]. 2021 Mar 11;11(1):5641. Available from: <http://dx.doi.org/10.1038/s41598-021-84269-8>)

**Supplemental Table 2. Location of infarctions on NeuroMix and cMRI, semi-automatic segmentation**

| **Location** | **Neuromix** | | **cMRI** | |
| --- | --- | --- | --- | --- |
|  | **Right** | **Left** | **Right** | **Left** |
|  |  |  |  |  |
| Frontal | 7 | 4 | 6 | 4 |
| Temporal | 3 | 2 | 3 | 2 |
| Parietal | 4 | 4 | 5 | 3 |
| Occipital | 6 | 4 | 5 | 4 |
| Cerebellum | 7 | 10 | 7 | 10 |
| Brainstem | 8 | 7 | 7 | 7 |
| Deep gray matter | 7 | 10 | 9 | 10 |
| **Total number:** | 42 | 41 | 42 | 40 |
